# Coffee Intake as a Risk Indicator for Tooth Loss in Korean Adults

**DOI:** 10.1038/s41598-018-20789-0

**Published:** 2018-02-05

**Authors:** In-Seok Song, Kyungdo Han, Jae-Jun Ryu, Yeon-Jo Choi, Jun-Beom Park

**Affiliations:** 1 Department of Oral and Maxillofacial Surgery, Korea University Anam Hospital, Seoul, Republic of Korea; 2 Department of Biostatistics, College of Medicine, The Catholic University of Korea, Seoul, Republic of Korea; 3 Department of Prosthodontics, Korea University Anam Hospital, Seoul, Republic of Korea; 4 Department of Periodontics, College of Medicine, The Catholic University of Korea, Seoul, Republic of Korea

## Abstract

The aim of this study was to examine the association between coffee intake and tooth loss. This study hypothesized that the intake of coffee would increase the prevalence of tooth loss in Korean adults. Subject information was obtained from the Korea National Health and Nutrition Examination Survey conducted in 2010–2011. Sociodemographic and lifestyle variables, anthropometric and biochemical status, metabolic health and glucose tolerance status, as well as oral health behaviors were evaluated. The number of remaining teeth was negatively associated with the frequency of coffee intake (*p*-value < 0.05). Daily coffee consumers had significantly higher levels of body mass index (BMI), waist circumference (WC), total cholesterol, and low density lipoprotein cholesterol (LDL-C) (all *p*-value < 0.05). Individuals with less than 20 remaining teeth had higher BMI, WC, diastolic blood pressure, and LDL-C (all *p*-value < 0.05). Finally, participants who drank coffee on a daily basis were more likely to have fewer remaining teeth. The prevalence of having less than 20 remaining teeth was 69% higher in groups with daily coffee intake than those with coffee intake of less than once a month after adjustment for potential covariates (Odds Ratio [95% CI] = 1.69 [1.35, 2.13]). In conclusion, daily coffee consumption is closely associated with tooth loss in Korean adults.

## Introduction

Coffee is one of the most popular brewed beverages and is derived from roasted coffee beans^1^. Coffee is composed of caffeine, chlorogenic acid, trigonelline, diterpenoids, cafestol, and kahweol^2^. Coffee has been shown to have beneficial effects for mood improvement, cognitive behavior, and endurance throughout extensive exercise^3,4^. Daily coffee consumption (>2 cups) among Korean adults^5^ increased by 48% from 2001 to 2011.

Coffee consumption has been reported to be associated with the prevention of several diseases. In the United States, a prospective cohort study revealed that coffee consumption was associated with lower mortality due to cardiovascular disease, chronic respiratory diseases, pneumonia and influenza, and self-injury, but not cancer^6^. However, there is still controversy about coffee consumption’s effects on some metabolic disorders. Several reports showed that coffee consumption was inversely correlated with the occurrence of metabolic syndrome^7,8^, even though another study reported that the consumption of instant coffee with high sugar content might increase the risk of metabolic syndrome^9^. Cohort studies revealed that coffee consumption was inversely associated with type 2 diabetes^10,11^, whereas, other reports demonstrated that coffee had no effect on insulin sensitivity or protection against diabetes^12,13^.

Tooth loss is a common problem that is especially found in the elderly. Tooth loss is caused by dental caries, periodontal disease, and trauma^14,15^. Tooth loss leads to decreased nutritional intake and general weakness which is linked to several medical problems including physical limitations and cognitive impairment^16^. A review pointed out that tooth loss clearly restricted nutritional and oral health, overall quality of life, and eventually shortened life expectancy^17^. Recently, a cross-sectional research revealed that periodontitis may be associated with coffee consumption^1^. However, another study demonstrated that coffee consumption had beneficial effects on periodontal health^18^.

Therefore, this study aimed to investigate the possible association between coffee consumption and tooth loss among Korean adults. We hypothesized that coffee intake would be associated with the occurrence of tooth loss.

## Materials and Methods

### Overview of the survey and participants

The data for this study were obtained from the 2010–2011 Korea National Health and Nutrition Examination Survey, a cross-sectional and nationwide survey supervised by the Ministry of Health and Welfare of South Korea. Specially trained investigators inspected a representative population of South Korean adults with well-designed questionnaires including physical inspections, health interviews, and nutritional examinations^19^.

**Figure 1:**
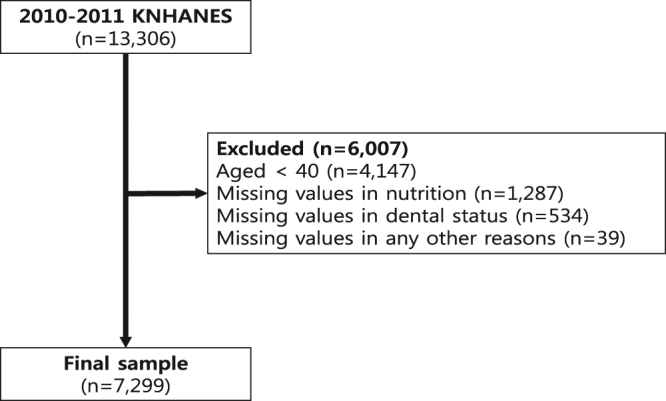
Flowchart of the study according to STROBE guidelines.

Initially, 13,306 participants were included in the study. 4,147 participants aged <40, 1,287 participants who had missing values in nutrition, 534 participants who had missing values in dental status, and 39 participants with any other missing values were removed from the study. A final total of 7,299 participants were selected for this study. All the participants provided written informed consent. This study was approved by the Institutional Review Board (IRB) of the Korean Center for Disease Control and Prevention, and was accomplished according to the Ethical Principles for Medical Research Involving Human Subjects based on the Helsinki Declaration. This study was confirmed according to the STROBE guidelines and presented as a flowchart (Fig. 1).

### Sociodemographic and lifestyle variables

Sociodemographic and lifestyle variables of the participants were collected with a self-administered questionnaire including those for alcohol drinking, cigarette smoking, household income, physical exercise, and education level. Smokers were categorized as ex-smokers, non-smokers, or current smokers. Alcohol users were categorized as heavy drinkers (>30 g/day), mild to moderate drinkers (1–30 g/day), or non-drinkers^20^. The level of education was classified as either having graduated from high school (≥13 years) or not. The rate of physical exercise was measured based on the International Physical Activity Questionnaire. Participants who performed body exercises for 30 minutes/session at least 5 times/week, or those who engaged in physical exercise for 20 minutes/session at least 3 times/week were categorized as regular exercisers. Household income was divided into quartiles by the amount earned by family members. Stress level was categorized as either “uncontrollable”, “stressful”, “controllable”, or “never”^21^. The place of residence was subdivided into urban and rural classifications. The presence of a spouse was also considered in the analysis.

### Anthropometric measurements

Qualified trained examiners evaluated the data. Height was measured to the nearest 0.1 cm, and body weight was documented using a digital scale to the nearest 0.1 kg in bare feet and lightweight clothing. Waist circumference was assessed to the nearest 0.1 cm at the slimmest mid-point between the costal and the iliac crest margins, over loose clothing at the end of a normal expiration^22^. BMI was measured by dividing body weight (kg) by the square of height (m^2^). Hypertension was defined as a systolic and/or a diastolic blood pressure that was consistently higher than 140 and 90 mm Hg, respectively. Pre-hypertension was categorized as elevated blood pressure above normal, but below hypertension as follows: a diastolic pressure 80–89 mm Hg or a systolic blood pressure 120–139 mm Hg. Neither hypertension nor pre-hypertension was categorized as normotension.

### Biochemical measurements

Trained personnel collected biochemical samples from the participants. A standard mercury sphygmomanometer (Baumanometer, W. A. Baum Co., Copiague, NY, USA) was used to calculate blood pressure. Systolic and diastolic blood pressures were checked three times at five minute intervals and an average was determined. Blood samples were obtained from the antecubital vein after an eight hour fast for each participant. Samples were stored immediately at −70 °C and then conveyed to a central testing institute (NeoDin Medical Institute, Seoul, South Korea). Low and high-density lipoprotein cholesterol, serum fasting plasma glucose (FPG), total cholesterol, and triglycerides were measured with an automated enzymatic analyzer (Hitachi 7600; Hitachi, Ltd., Tokyo, Japan).

### Descriptions of metabolic syndrome

Metabolic syndrome in Asians was defined according to American Heart Association/National Heart, Lung, and Blood Institute criteria^23^. Participants with at least three of the following 5 medical conditions were defined as having metabolic syndrome: waist circumference ≥ 80 cm for women and ≥90 cm for men, current use of an anti-hypertensive drug or blood pressure ≥130/85 mmHg, use of an anti-diabetic drug or FPG ≥ 100 mg/dL, use of an anti-dyslipidemic drug or fasting triglycerides ≥ 150 mg/dL, and use of an anti-dyslipidemic drug or high-density lipoprotein cholesterol < 50 mg/dL in women and <40 mg/dL in men.

### The number of remaining teeth and oral health behaviors

The number of remaining teeth was examined by the trained dental staff member who were under license. Participants reported the frequency of daily tooth brushing, which was defined as the total number of tooth brushing sessions per day. Self-reported oral health status and number of dental visits within a year were also recorded^24^. Self-reported oral health status was categorized as “good”, “moderate”, or “bad”. Dental pain within the previous year was defined as whether the participant felt pain or discomfort in their dental area during that year.

### Statistical analyses

The data are expressed as a percentage (standard error) for categorical variables and mean ± standard error for continuous variables. Rao–Scott Chi-square tests for categorical variables and Student’s t-tests for continuous variables were used. A multiple linear regression analysis was performed examining the frequency of coffee consumption and biochemical parameters after adjustment for covariates including age and gender. The association between number of remaining teeth and frequency of coffee consumption was analyzed using one-way analysis of covariance (ANCOVA). Finally, multiple logistic regression analyses were conducted to determine the odd ratios for having less than 20 remaining teeth according to the frequency of coffee consumption. Model 1 was a model with no adjustment. Model 2 was adjusted for sociodemographic covariates including gender and age. Model 3 was adjusted for components of Model 2 and lifestyle variables including smoking, drinking, physical activity, BMI, education status, and household income. Model 4 was adjusted for the components of Model 3 plus diseases and dental variables (metabolic syndrome, stress level, and number of daily tooth brushing sessions). The SAS statistical software version 9.3 (SAS institute, Cary, NC, USA) was used for all analyses. Statistical significance was defined as *p* < 0.05.

## Results

Table 1 displays the characteristics of the included participants. The participants were divided into two groups by the number of remaining teeth (≥20 or <20). The prevalence of having less than 20 remaining teeth was significantly higher in individuals with high waist circumference, systolic blood pressure, serum triglyceride, and household income in the lowest quartile. Individuals with metabolic syndrome were more likely to have less than 20 remaining teeth (all *p*-values < 0.05).

**Table 1:** Participant characteristics.

	Remaining teeth (n) < 20		
	No	Yes	*p*-value
n	5359	1940	
Age (year)	52.5 ± 0.2	66.6 ± 0.37	<0.001
Gender (M)	48.3 (0.7)	46.4 (1.5)	0.31
BMI (kg/m^2^)	24.1 ± 0.05	23.6 ± 0.09	<0.001
BMI ≥ 25 (yes)	36.5 (0.8)	32.2 (1.2)	0.03
WC (cm)	82.7 ± 0.2	83.6 ± 0.27	0.01
SBP (mmHg)	121.9 ± 0.32	129.3 ± 0.61	<0.001
DBP (mmHg)	79 ± 0.21	76.5 ± 0.36	<0.001
FPG (mg/dL)	99.7 ± 0.42	104.6 ± 0.86	<0.001
TC (mg/dL)	194.3 ± 0.65	192.6 ± 1.12	0.18
HDL-C (mg/dL)	52.3 ± 0.23	50.6 ± 0.39	<0.001
LDL-C (mg/dL)	114.7 ± 0.59	113.5 ± 0.92	0.26
TG* (mg/dL)	118 (115.4–120.6)	125 (120.2–130)	0.01
Present smoker (yes)	20 (0.8)	21.4 (1.3)	0.34
Drinking alcohol monthly (yes)	55.7 (0.9)	40.3 (1.3)	<0.001
Regular physical exercise (yes)	22.2 (0.8)	17.8 (1.3)	0.02
Income (lowest quartile)	15.8 (0.8)	40.4 (1.6)	<0.001
Education level (>13years)	61.3 (1.1)	22.6 (1.4)	<0.001
Place of residence (urban)	76.8 (2.4)	61.5 (3.8)	<0.001
Presence of spouse (yes)	87.5 (0.6)	70.4 (1.6)	<0.001
MetS (yes)	33.2 (0.8)	48 (1.6)	<0.001
WC_Mets (yes)	39.3 (0.9)	45.8 (1.3)	<0.001
Daily tooth brushing (n)			<0.001
≤1	12.5 (0.7)	23.2 (1.4)	
2	50.9 (1)	48.6 (1.5)	
≥3	36.6 (1.1)	28.2 (1.7)	
Dental visit within a year (yes)	25.9 (1)	15.1 (1.3)	<0.001
Self-reported oral health status(yes)			<0.001
1	14.4 (0.7)	7.8 (0.8)	
2	40.7 (1)	20.5 (1.5)	
3	44.9 (0.9)	71.7 (1.7)	
Dental pain within a year (yes)	27.7 (1)	25 (1.6)	0.12

Data are presented as mean ± standard error for continuous variables and percentage (standard error) for categorical variables. *P-values were obtained by independent t-tests for continuous variables or Chi-square tests for categorical variables. *Geometric mean (95% CI). Hypertension was defined as >140/90 mmHg, prehypertension as the systolic blood pressure 120–139 mm Hg or a diastolic pressure 80–89 mm Hg. Neither hypertension nor prehypertension were designated as normotension. Self-reported oral health statuses were divided into score 1 as good, 2 as moderate, and 3 as bad. Abbreviation: BMI; body mass index, WC; waist circumference, SBP, systolic blood pressure; DBP, diastolic blood pressure; FBG, fasting blood glucose; TC, total cholesterol; HDL-C, high density lipoprotein cholesterol; LDL-C, low density lipoprotein cholesterol; TG, triglyceride, Mets; metabolic syndrome, WC_Mets; waist circumference which met the inclusion criteria for metabolic syndrome, WC ≥ 90 cm for men, and ≥80 cm for women.

Table 2 shows the associations between the number of remaining teeth, the frequency of coffee intake, and biochemical/anthropometric parameters by one-way ANCOVA which was adjusted for covariates including age and sex. Daily coffee consumers had significantly higher levels of body mass index (BMI), waist circumference (WC), total cholesterol (TC), and low density lipoprotein cholesterol (LDL-C) (all *p*-value < 0.05) after adjustment for age and gender. Similarly, individuals with less than 20 remaining teeth had significantly higher levels of BMI, WC, diastolic blood pressure (DBP), and LDL-C (all *p*-value < 0.05).

**Table 2:** Association between the number of remaining teeth, coffee intake, and biochemical/anthropometric parameters.

	BMI	WC	SBP	DBP	FPG	TC	HDL-C	LDL-C	TG*
**Coffee intake** (**n**)									
<1/mo	23.7 ± 0.1	82.3 ± 0.3	125.1 ± 0.6	77.7 ± 0.4	102.8 ± 1.4	191.3 ± 1.6	51.1 ± 0.5	111.2 ± 1.3	125 (118.3–132)
2/mo–1/wk	23.9 ± 0.2	83.3 ± 0.6	125 ± 1	77.9 ± 0.6	100.6 ± 1	191.4 ± 1.7	52.3 ± 0.8	111.2 ± 1.5	122.6 (114.7–130.9)
2–6/wk	23.7 ± 0.1	82.3 ± 0.4	124.6 ± 0.7	77.1 ± 0.5	103.1 ± 1.2	193 ± 1.6	53 ± 0.6	114.1 ± 1.5	116.4 (111.1–121.9)
Daily	24.1 ± 0.1	83.2 ± 0.2	125.2 ± 0.3	77.9 ± 0.2	100.6 ± 0.4	195.7 ± 0.7	51.9 ± 0.2	116.6 ± 0.6	117.5 (114.8–120.3)
*p*-value	0.01	0.01	0.9	0.36	0.19	0.01	0.11	<0.001	0.1
**Remaining teeth** (**n**)									
<20	24.1 ± 0.1	83.1 ± 0.2	125.1 ± 0.3	78.1 ± 0.2	100.6 ± 0.5	195.2 ± 0.7	52 ± 0.2	115.9 ± 0.6	118.2 (115.6–120.8)
20	23.6 ± 0.1	82.5 ± 0.3	125.1 ± 0.6	77 ± 0.4	102.8 ± 0.9	192.6 ± 1.2	51.5 ± 0.4	113.1 ± 1	120.4 (115.4–125.8)
*p*-value	**<**0.001	0.04	0.97	0.02	0.06	0.06	0.3	0.02	0.47

*Geometric mean (95% CI). *p-value < 0.05 designated statistical significance. Data were analyzed by one-way analysis of covariance (ANCOVA), and adjusted for covariates including age and gender. Abbreviations: WC; waist circumference, BMI; body mass index, SBP; systolic blood pressure, DBP; diastolic blood pressure, FPG; fasting plasma glucose, TC; total cholesterol, HDL-C; high density lipoprotein cholesterol, LDL-C; low density lipoprotein cholesterol, TG; serum triglyceride.

Table 3 represents the mean number of remaining teeth according to the frequency of coffee intake by one-way ANCOVA. The mean number of remaining teeth among participants were negatively associated with the frequency of coffee intake (all *p*-value < 0.05).

**Table 3:** The number of remaining teeth according to the frequency of coffee intake.

	Remaining teeth (n)			
	Model 1	Model 2	Model 3	Model 4
**Coffee intake (n)**				
<1/mo	21.9 ± 0.3	22.3 ± 0.2	22.4 ± 0.2	23 ± 0.2
2/mo–1/wk	22.4 ± 0.4	22.3 ± 0.3	22.3 ± 0.3	22.9 ± 0.3
2–6/wk	22.4 ± 0.3	21.9 ± 0.2	22.1 ± 0.2	22.6 ± 0.2
daily	23.3 ± 0.2	21.6 ± 0.1	21.7 ± 0.1	22.2 ± 0.1
*p*-value	<0.001	0.02	0.02	0.004

The data are presented as mean ± standard error for continuous variables. *p-value < 0.05 designated statistical significance. Data were analyzed by one-way ANCOVA. MODEL1 was non-adjusted. MODEL2 was adjusted for gender and age. MODEL3 was adjusted for gender, age, drinking, smoking, household income, physical exercise, and education level. MODEL4 was adjusted for gender, age, drinking, smoking, metabolic syndrome, household income, physical exercise, education level, BMI, number of daily tooth brushing sessions, and stress level.

Table 4 shows the prevalence of having less than 20 remaining teeth according to the frequency of coffee intake. The prevalence of having less than 20 remaining teeth was about 69% higher in daily coffee consumers than individuals having coffee less than one time per month after adjustment for covariates (Odds Ratio [95% Confidence Intervals] = 1.69 [1.35, 2.13]).

**Table 4:** Prevalence of having less than 20 remaining teeth according to the frequency of coffee intake

	OR (95% CI)			
	Model 1	Model 2	Model 3	Model 4
**Coffee intake (n)**				
<1/mo	1 (ref.)	1 (ref.)	1 (ref.)	1 (ref.)
2/mo–1/wk	0.87 (0.64, 1.17)	1.02 (0.73, 1.42)	1.07 (0.78, 1.48)	1.16 (0.81, 1.64)
2–6/wk	0.91 (0.73, 1.14)	1.19 (0.91, 1.55)	1.18 (0.90, 1.54)	1.34 (1.02, 1.78)
daily	0.70 (0.59, 0.83)	1.43 (1.15, 1.77)	1.48 (1.19, 1.84)	1.69 (1.35, 2.13)

Multiple logistic regression analyses were performed. MODEL1 was non-adjusted. MODEL2 was adjusted for gender and age. MODEL3 was adjusted for gender, age, drinking, smoking, household income, physical exercise, and education level. MODEL4 was adjusted for gender, age, drinking, smoking, metabolic syndrome, household income, physical exercise, education level, BMI, number of daily tooth brushing sessions, and stress level.

Finally, the prevalence of having less than 20 remaining teeth by age group were assessed (the elderly 65 or more vs. others). However, we did not find any association between age group (Supplementary information).

## Discussion

This study found that daily coffee consumers had significantly higher levels of BMI, WC, TC, and LDL-C. Individuals with metabolic syndrome or its components were more likely to have less than 20 remaining teeth. The mean number of remaining teeth among participants was negatively associated with the frequency of coffee intake. Finally, the prevalence of having less than 20 remaining teeth was 69% higher in daily coffee consumers than those who had coffee less than one time per month after adjustment for covariates.

This study found that coffee consumption may have harmful effects on dental health, leading to tooth loss. An increasing trend of instant coffee consumption in South Korea may explain the relationship between coffee consumption and tooth loss^5^. A cross-sectional study among Korean adults showed that 76% of the participants were habitual coffee drinkers, most of whom consumed instant coffee mixed with sugar and powdered creamer^9^. The study showed that instant coffee consumers had an elevated risk of metabolic syndrome and its components including obesity, abdominal obesity, and hypo-HDL-C. Other studies reported that the consumption of instant coffee with high sugar content may increase the risk of metabolic syndrome^9^. A qualitative systemic review also showed that sugar from instant coffee-mix contributed to increased HDL-C and risk of metabolic syndrome^25^. Since several studies demonstrated that metabolic syndrome is closely associated with tooth loss^26–29^, one can expect that the habitual consumption of a sugar containing coffee mix could increase tooth loss.

People who drink coffee can add sugar or syrup to their coffee which can cause tooth structure damage^30^. Women who drank sweetened coffee either 1–4 or >5 cups per day had higher risk for tooth loss. Sugar contained in coffee may lead to bacterial fermentation which can result in the destruction of the enamel surface^31^. Similarly, other studies revealed that caries risk increased in cases of coffee consumption with additives including sweeteners and creaming agents, whereas coffee consumption without additives had a caries preventive effect^32^. They also found that the caries index was 2.9 in individuals who drank black coffee and 5.5 for individuals who drank coffee with additives.

Individuals with periodontitis demonstrated significantly more coffee consumption in Korean male adults. They had a 45% increased prevalence of periodontitis when they consumed coffee 3 or more times per day^1^. Experimental studies supported this finding. In rodents, the daily consumption of high doses of caffeine provoked ligature-induced periodontitis^33,34^. Since periodontitis is one of the leading causes of tooth loss, coffee consumption may lead to tooth loss by periodontal breakdown.

Coffee/caffeine consumption may lead to a malfunction in calcium metabolism, reduction in bone mineral density, and delayed bone repair^35^. Caffeine may inhibit the development of osteoblasts by decreasing the expression of vitamin D receptors on the surface of osteoblasts^36^, or by causing the upstream mediator cyclic AMP to down-regulate osteoblast proliferation^37^. Another experimental study showed that daily caffeine consumption may increase osteoclastogenesis^38^. The intake of coffee was significantly associated with an increased risk for osteoporosis and osteoporotic fracture^39^. Since caffeine can provoke osteoporosis, tooth loss also increased as osteoporosis risk increased^40^. Coffee consumption may elevate calcium excretion through urine which may also increase osteoporosis^41^. Calcium loss may be harmful to the elderly who have less calcium intake. Recent long-term longitudinal cohort studies revealed that a high coffee intake of 4 or more cups daily was associated with a small reduction in bone density^42^.

Collectively, one can assume that sugar and other ingredients from instant coffee mix may increase the risk of metabolic syndrome and its components. In addition, dietary carbohydrates from sugar components may increase the risk of dental caries or periodontal disease, which eventually lead to tooth loss. Looking from a different point of view, the long-term intake of coffee may evoke catabolic bone metabolism, decreasing the density of alveolar bone, and eventually leading to tooth loss.

This study has some limitations. First, this study cannot clarify the precise causal relationship between tooth loss and coffee consumption because of its cross-sectional observational design. Further longitudinal cohort studies are necessary to clarify the precise effect of coffee consumption on tooth loss. Second, this study did not subdivide the sample by age group, brand, or type of coffee, which could affect the outcome variables. Third, either coffee volume or caffeine content could vary among different size of cups, which might influence the reliability of the study. Fourth, this study didn’t deal with effect of coffee with additives like sugar, milk or cream, which may have different effects on metabolism. This study also has strengths. First, to the best of our knowledge, this is the first study that demonstrated the close association between coffee consumption and tooth loss in South Korean adults. Second, the present study showed that oral and metabolic health could benefit from less coffee consumption. The findings from this study emphasize the oral health issues of coffee consumption as well as metabolic health.

## Conclusion

Daily coffee consumption is associated with tooth loss in Korean adults.

## Electronic supplementary material

Supplementary Information
