# Supplementary material for: Coffee Intake as a Risk Indicator for Tooth Loss in Korean Adults
Source: Sci Rep. 2018 Feb 5;8:2392. doi: 10.1038/s41598-018-20789-0 (PMC5799212; doi:10.1038/s41598-018-20789-0)
Supplement: Supplementary file 1 — Supplementary Information [file 41598_2018_20789_MOESM1_ESM.pdf]

Coffee Intake as a Risk Indicator for Tooth Loss in Korean Adults

In-Seok Song DDS MSD PhD[1], **Kyungdo Han** PhD[2], **Jae-Jun Ryu** DDS MSD PhD[3], **Yeon-Jo Choi** DMD MS PhD[3*], **Jun-Beom Park** DDS MSD PhD[4*]

[1]Department of Oral and Maxillofacial Surgery, Korea University Anam Hospital, Seoul, Republic of Korea

[2]Department of Biostatistics, College of Medicine, The Catholic University of Korea, Seoul, Republic of Korea

[3]Department of Prosthodontics, Korea University Anam Hospital, Seoul, Republic of Korea

[4]Department of Periodontics, College of Medicine, The Catholic University of Korea, Seoul, Republic of Korea

*These two authors contributed equally to this work.

Address correspondence to:

Jun-Beom Park DDS MSD PhD

Department of Periodontics, Seoul St Mary's Hospital, College of Medicine, The Catholic University of Korea, 222, Banpo-daero, Seocho-gu, Seoul, 16591, Republic of Korea, Tel: +82-10-4325-2651; E-mail: jbassoonis@yahoo.co.kr

Yeon-Jo Choi DMD MS PhD

Department of Prosthodontics, Anam Hospital, Korea University, 73 Inchon-ro, Seongbuk- gu, Seoul, Republic of Korea, 02841, Tel:+82-10-7225-8988, E-mail: yeonjochoi@yahoo.co.kr

Running Head: Coffee and tooth loss

Prevalence of having less than 20 remaining teeth by age group (the elderly 65 or more vs. others).

	Model1		Model2		Model3		Model4	
	<65	65<=	<65	65<=	<65	65<=	<65	65<=
**Coffee intake (n)**								
**< 1/mo**	1	1	1	1	1	1	1	1
**2/mo - 1/wk**	1.03(.60,1.77)	1.1(.753,1.61)	.89(.51,1.55)	1.11(.76,1.62)	.9(.52,1.54)	1.22(.84,1.77)	.98(.54,1.75)	1.37(.93,2.01)
**2-6/wk**	1.2(.79,1.81)	1.08(.79,1.49)	1.21(.79,1.86)	1.15(.83,1.58)	1.15(.74,1.79)	1.18(.85,1.64)	1.4(.87,2.26)	1.28(.92,1.79)
**daily**	1.13(.81,1.58)	1.24(.97,1.59)	1.38(.98,1.95)	1.43(1.1,1.86)	1.41(1.0,1.99)	1.49(1.13,1.95)	1.73(1.22,2.47)	1.61(1.2,2.15)
**P for interaction**	.85		.57		.52		.77	

Multiple logistic regression analyses were performed. MODEL1 was non-adjusted. MODEL2 was adjusted for gender and age. MODEL3 was adjusted for gender, age, drinking, smoking, household income, physical exercise, and education level. MODEL4 was adjusted for gender, age, drinking, smoking, metabolic syndrome, household income, physical exercise, education level, BMI, number of daily tooth brushing sessions, and stress level.

STROBE Statement—checklist of items that should be included in reports of observational studies

	Item No	Recommendation	Page number
**Title and abstract**	1	(*a*) Indicate the study's design with a commonly used term in the title or the abstract	3
		(*b*) Provide in the abstract an informative and balanced summary of what was done and what was found	3
**Introduction**			
Background/rationale	2	Explain the scientific background and rationale for the investigation being reported	5
Objectives	3	State specific objectives, including any prespecified hypotheses	5
**Methods**			
Study design	4	Present key elements of study design early in the paper	6
Setting	5	Describe the setting, locations, and relevant dates, including periods of recruitment, exposure, follow-up, and data collection	6
Participants	6	(*a*) *Cohort study*—Give the eligibility criteria, and the sources and methods of selection of participants. Describe methods of follow-up<br>*Case-control study*—Give the eligibility criteria, and the sources and methods of case ascertainment and control selection. Give the rationale for the choice of cases and controls<br>*Cross-sectional study*—Give the eligibility criteria, and the sources and methods of selection of participants	6
		(*b*) *Cohort study*—For matched studies, give matching criteria and number of exposed and unexposed<br>*Case-control study*—For matched studies, give matching criteria and the number of controls per case	

Variables	7	Clearly define all outcomes, exposures, predictors, potential confounders, and effect modifiers. Give diagnostic criteria, if applicable	6-10
Data sources/ measurement	8*	For each variable of interest, give sources of data and details of methods of assessment (measurement). Describe comparability of assessment methods if there is more than one group	6-10
Bias	9	Describe any efforts to address potential sources of bias	6
Study size	10	Explain how the study size was arrived at	6
Quantitative variables	11	Explain how quantitative variables were handled in the analyses. If applicable, describe which groupings were chosen and why	6
Statistical methods	12	(*a*) Describe all statistical methods, including those used to control for confounding	10
		(*b*) Describe any methods used to examine subgroups and interactions	10
		(*c*) Explain how missing data were addressed	6
		(*d*) *Cohort study*—If applicable, explain how loss to follow-up was addressed<br>*Case-control study*—If applicable, explain how matching of cases and controls was addressed<br>*Cross-sectional study*—If applicable, describe analytical methods taking account of sampling strategy	6
		(*e*) Describe any sensitivity analyses	

Continued on next page

**Results**			
Participants	13*	(a) Report numbers of individuals at each stage of study—eg numbers potentially eligible, examined for eligibility, confirmed eligible, included in the study, completing follow-up, and analysed	6
		(b) Give reasons for non-participation at each stage	6
		(c) Consider use of a flow diagram	6
Descriptive data	14*	(a) Give characteristics of study participants (eg demographic, clinical, social) and information on exposures and potential confounders	10
		(b) Indicate number of participants with missing data for each variable of interest	10
		(c) *Cohort study*—Summarise follow-up time (eg, average and total amount)	
Outcome data	15*	*Cohort study*—Report numbers of outcome events or summary measures over time	
		Case-control study—Report numbers in each exposure category, or summary measures of exposure	
		Cross-sectional study—Report numbers of outcome events or summary measures	*10*
Main results	16	(*a*) Give unadjusted estimates and, if applicable, confounder-adjusted estimates and their precision (eg, 95% confidence interval). Make clear which confounders were adjusted for and why they were included	10
		(*b*) Report category boundaries when continuous variables were categorized	10
		(*c*) If relevant, consider translating estimates of relative risk into absolute risk for a meaningful time period	
Other analyses	17	Report other analyses done—eg analyses of subgroups and interactions, and sensitivity analyses	11
**Discussion**			
Key results	18	Summarise key results with reference to study objectives	12
Limitations	19	Discuss limitations of the study, taking into account sources of potential bias or imprecision. Discuss both direction and magnitude of any potential bias	14

Interpretation	20	Give a cautious overall interpretation of results considering objectives, limitations, multiplicity of analyses, results from similar studies, and other relevant evidence	13
Generalisability	21	Discuss the generalisability (external validity) of the study results	14
**Other information**			
Funding	22	Give the source of funding and the role of the funders for the present study and, if applicable, for the original study on which the present article is based	15

*Give information separately for cases and controls in case-control studies and, if applicable, for exposed and unexposed groups in cohort and cross-sectional studies.

Note: An Explanation and Elaboration article discusses each checklist item and gives methodological background and published examples of transparent reporting. The STROBE checklist is best used in conjunction with this article (freely available on the Web sites of PLoS Medicine at http://www.plosmedicine.org/, Annals of Internal Medicine at http://www.annals.org/, and Epidemiology at http://www.epidem.com/). Information on the STROBE Initiative is available at www.strobe-statement.org.
